# Surprising magic of CD24 beyond cancer

**DOI:** 10.3389/fimmu.2023.1334922

**Published:** 2024-01-19

**Authors:** He Wang, Peng Shi, Xinyu Shi, Yaqing Lv, Hongwei Xie, Hai Zhao

**Affiliations:** ^1^ Department of Neurosurgery, The Affiliated Hospital of Qingdao University, Qingdao, China; ^2^ Department of Emergency Surgery, The Affiliated Hospital of Qingdao University, Qingdao, China; ^3^ Department of Radiology, the Affiliated Hospital of Qingdao University, Qingdao, China; ^4^ Department of Outpatient, The Affiliated Hospital of Qingdao University, Qingdao, China

**Keywords:** CD24, CD47, innate checkpoint, non-neoplastic, autoimmune diseases, Covid-19, neurological disease, metabolic diseases

## Abstract

CD24 has emerged as a molecule of significant interest beyond the oncological arena. Recent studies have unveiled its surprising and diverse roles in various biological processes and diseases. This review encapsulates the expanding spectrum of CD24 functions, delving into its involvement in immune regulation, cancer immune microenvironment, and its potential as a therapeutic target in autoimmune diseases and beyond. The ‘magic’ of CD24, once solely attributed to cancer, now inspires a new paradigm in understanding its multifunctionality in human health and disease, offering exciting prospects for medical advancements.

## Introduction

CD24 is a glycosylphosphatidylinositol-anchored protein that was first identified in 1978 as a B-cell differentiation antigen (1). It is expressed in a wide range of tissues and cell types, including hematopoietic stem cells, B and T lymphocytes, epithelial cells, and neural cells (2–11). CD24 has been found to play a role in a variety of physiological and pathological processes, including cell adhesion, migration, differentiation, and apoptosis (12).

**Figure 1:**
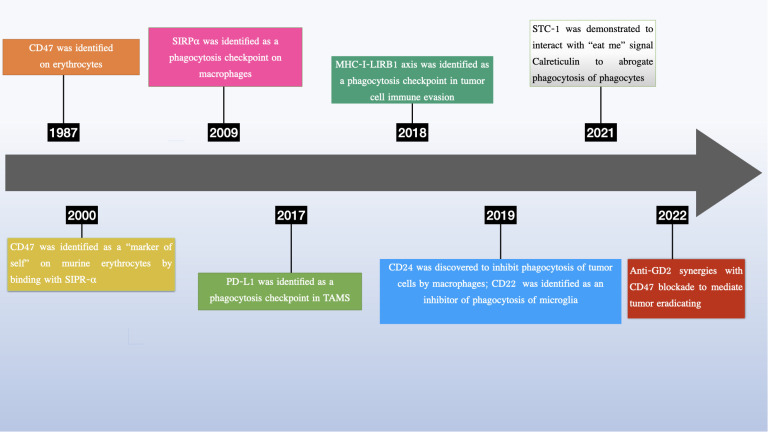
Discovery of phagocytosis checkpoints.

In recent years, there has been growing interest in phagocytosis checkpoints, especially CD24, as potential therapeutic targets for cancer treatment (13–15) (see **Figure 1**). Given the heterogeneity of in post-translational modifications, CD24 has been implicated in tumor growth, invasion, and metastasis, and has been suggested as a potential marker for cancer prognosis and therapy (14, 16–21). More importantly, growing research has uncovered vital functions of CD24 in a range of pathological states, such as autoimmune disorders (22–24), sepsis (25) metabolic disorders (26), graft vs host diseases (27).

In this review, we offer an in-depth examination of CD24, encompassing fundamental principles and pertinent pathways. We also emphasize the significance of CD24 in both cancer and non-neoplastic conditions. Furthermore, we shed light on the ongoing clinical progress in targeting CD24 and identify the obstacles and possible remedies within the realm of cancer immunotherapy and non-neoplastic disorders. Our objective is to not only advance our comprehension of the existing CD24 research landscape but also to delve into the prospects of CD24-based immunotherapeutic approaches.

## Structure of CD24

CD24 is expressed on various cell types, including immune cells, neural cells, and cancer cells, and it is a glycosyl-phosphatidylinositol (GPI)-anchored protein with distinct domains: an intracellular domain, a transmembrane domain, and a heavily glycosylated extracellular domain (2–11, 28, 29). The extracellular domain of CD24 has varying numbers of N-linked and O-linked glycosylation points, playing a role in controlling CD24-driven cell attachment and signal transmission (30). It has one N-glycosylation site and multiple O-glycosylation sites that play a role in its glycosylation. The protein is attached to the cell membrane using a glycosylphosphatidylinositol (GPI) anchor at its end (C-terminus) (30). The CD24 crystal structure was defined using X-ray crystallography, revealing a tight, spherical shape with a β-barrel configuration made of 4 antiparallel β-strands. A disulfide bond between Cys53 and Cys73 provides stability to the β-barrel. The beginning section of the protein showcases a brief α-helix and an adaptable loop area (31).

## CD24 and its receptors

CD24 is a cell surface protein that interacts with a range of cell surface receptors, such as P-selectin, Siglec-10, and β1 integrin, and is involved in regulating cell adhesion, migration, cell differentiation, and apoptosis through its association with the Notch signaling pathway (15, 32–34).

**Figure 2:**
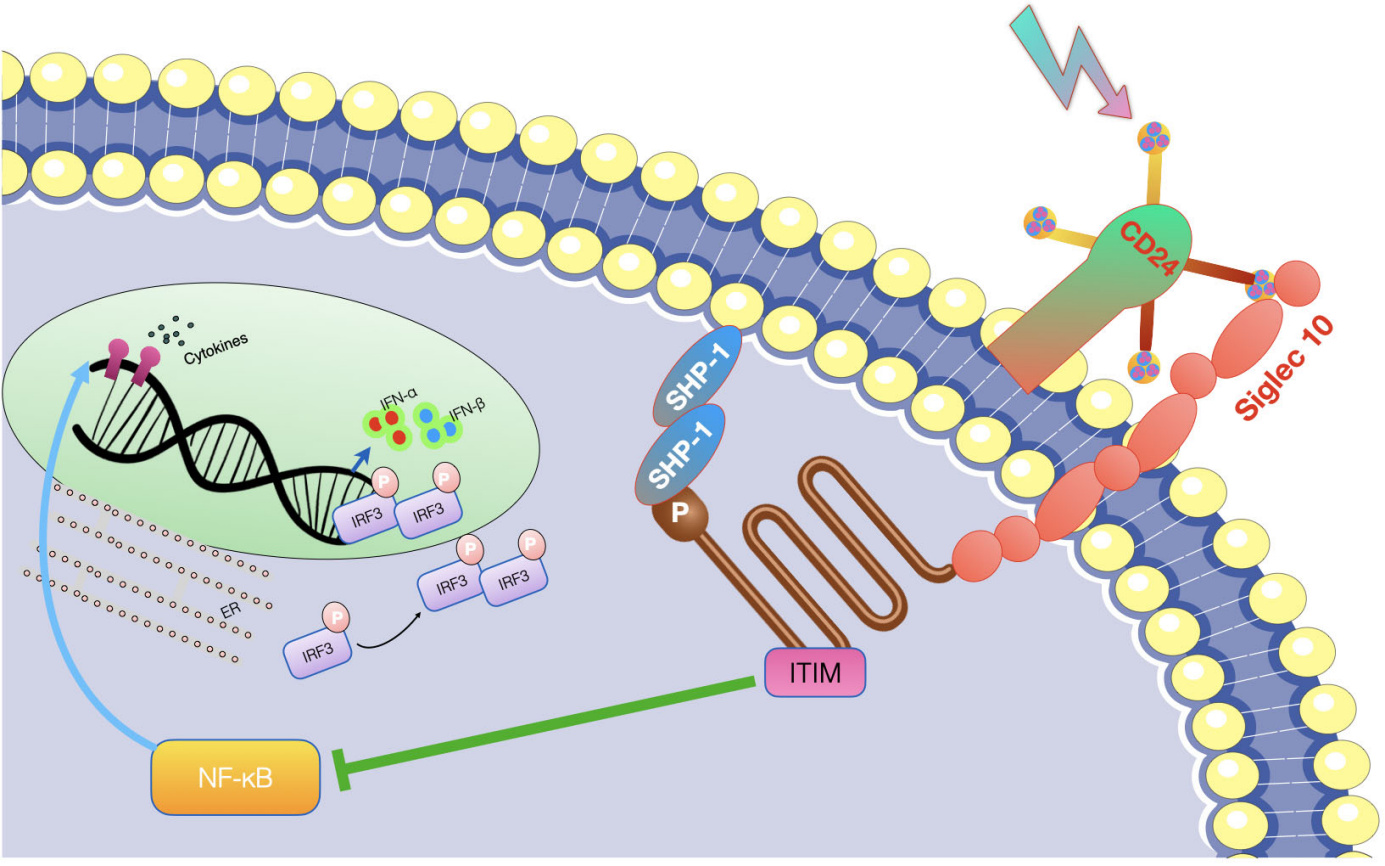
CD24-Siglec-10 pathway as hot targets for tumor immunotherapy. The CD24 antibody has the potential to enhance the immune system’s ability to eliminate tumors. By inhibiting CD24 molecule present on the surface of tumor stem cells, CD24 antibody can block the CD24-Siglec-g/10 signal activation, allowing macrophages to recognize and eliminate tumor cells through the immune clearance process.

While CD24 lacks intrinsic enzymatic activity, it interacts with multiple receptor proteins, such as Siglec-10, Siglec-15, and the NKG2D receptor (35, 36). The Siglec family of receptors, found on immune cells, engage with CD24 in a sialic acid-dependent manner. Siglec-10 functions as a negative regulator of immune responses and inhibits dendritic cells and B cells’ activation when interacting with CD24 (15, 37, 38). Siglec-15, expressed on osteoclasts, interacts with CD24, influencing osteoclast differentiation and bone resorption (39–41). The NKG2D receptor, present on natural killer cells and other immune cells, recognizes stress-induced ligands on tumor and infected cells. CD24 acts as a ligand for NKG2D, suppressing NKG2D-mediated immune responses and facilitating tumor immune evasion (13, 42) (see **Figure 2**).

In conclusion, CD24 is a cell surface protein involved in critical interactions with various receptors, impacting immune regulation, bone health, and tumor immune evasion. Understanding these CD24 functions and its interactions with receptors is essential for developing CD24-targeted therapies for various diseases.

## Role of CD24 in cancer

Studies have shown that CD24 is highly expressed in various tumor cells (21, 43). Recent studies have demonstrated that increased CD24 levels in the blood might be a new prognostic indicator and a biomarker for early cancer detection (18, 44–47). Mechanisms of tumorigenesis promotion by CD24 include cancer stem cell regulation, metastasis of tumor cell, proliferation of cancer cells and evasion of immune detection.

**Figure 3:**
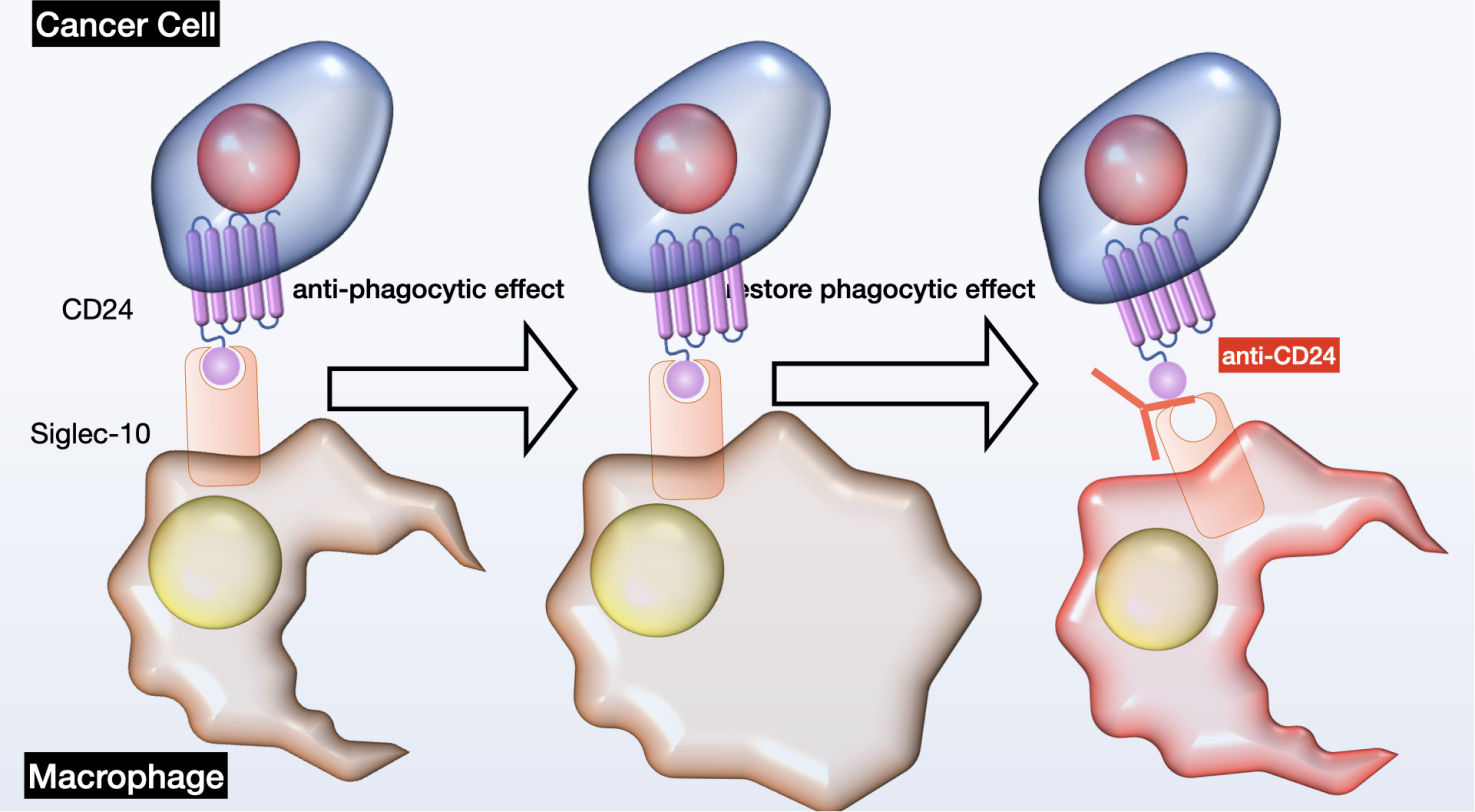
Schematic of CD24-Siglec-10 signaling in cancer immunotherapy. This diagram depicts the inhibitory receptor Siglec-10 identifying and binding to its ligand CD24 on ovarian cancer cells, leading to anti-phagocytic signaling pathways. Intervening with CD24 could potentially reinstate immune cell phagocytosis. OC, ovarian cancer; Siglec, sialic-acid-binding Ig-like lectin.

Initially, scientists have introduced the idea of cancer stem cells as the originating precursor cells in the formation of tumors. The association between CD24 and cancer stem cells has been proved in various types of cancer, including breast cancer (48), ovarian cancer (49), pancreatic cancer (50), hepatocellular carcinoma (51), bladder cancer (52), melanoma (53), colon cancer (54), leukemia (55), and multiple myeloma (56). CD24 expression was linked to CSC-like characteristics and the tumorigenic potential of these cells, suggesting that CD24 could serve as a surface marker for CSCs in both solid and hematological tumors; however, the cellular mechanisms of the CD24 - mediated effects are still unclear. Secondly, CD24 plays a crucial role in promoting tumor cell metastasis via multiple mechanisms. By attaching to P-selectin, it diminishes the adhesion of tumor cells, which aids in their movement along the vascular endothelium and platelets, thus increasing their ability to migrate and metastasize (57). CD24 also plays a part in the E-selectin-mediated movement of tumor cells across the surface of vascular endothelium, and it activates integrin subunits, enabling tumor cells to bind to extracellular matrix components and increase their mobility (58). Additionally, CD24 regulates key factors like STAT3 and tissue factor pathway inhibitor-2 (TFPI-2), influencing tumor metastasis by affecting their expression and activity (59, 60). Thirdly, CD24 significantly affects the growth of tumor cells by altering the expression of crucial signaling molecules. Microarray analysis has shown that CD24 mAb downregulates genes associated with carcinogenesis, including MAPK, Ras, and Bcl-2 (61). Additionally, CD24 is capable of initiating ERK and p38MAPK activation, which stimulates the proliferation of tumor cells in both controlled lab environments and living beings. CD24 also regulates the epidermal growth factor receptor (EGFR), a critical player in cell proliferation, by inhibiting its internalization and degradation, thereby affecting cell proliferation (62). Furthermore, the anti-CD24 mAb, G7 mAb, enhances the inhibitory effect of cetuximab on tumor proliferation *in vivo*, suggesting that CD24 might promotes the growth of tumor cells through the regulation of EGFR expression (63). Fourthly, CD24 plays a critical role in promoting tumor immune evasion through its interaction with Siglec-10. Siglec-10 binds tightly to CD24 in a sialic acid-dependent manner, leading to the inhibition of macrophage signaling cascades and diminished efficiency of phagocytosis, ultimately enhancing tumor immune escape (15). Additionally, elevated expression of Siglec-10 on natural killer (NK) cells correlates with weakened NK cell activity, further making tumors evade the immune system (64). CD24 has been demonstrated to trigger cell death in B cells and precursor B acute lymphoblastic leukemia cells, potentially impacting cellular immunity (65). Siglec-10 inhibits T cell activation, and malignant tumor-derived extracellular vesicles can upregulate Siglec-10 expression in T cells inside tumor microenvironment, decreasing T cell activation. Moreover, correctly glycosylated CD24 can bind to Siglec-10, blocking the activation of T cell receptors by suppressing kinases associated with TCR. These interactions collectively contribute to tumor immune evasion (66, 67) (see **Figure 3**).

CD24 plays a pivotal role in promoting tumor growth, metastasis, and immune evasion. Experimental investigations involving the deletion of the CD24 gene and therapeutic interventions have shown significant inhibition of tumor growth in animal models and improved patient survival (15). Consequently, targeting CD24 emerges as a promising therapeutic strategy for cancer treatment. However, it’s noteworthy that the creation of targeted treatments focusing on CD24 is mainly at the preclinical research phase, and comprehensive clinical trial data are currently limited.

To date, there have been two completed clinical trials focused on testing CD24-blocking drugs in cancer patients. The initial trial was a combined Phase 1/2 study involving 58 patients suffering from aggressive B cell lymphoproliferative disorders following bone marrow or organ transplants (68, 69). The participants in the study were administered a dual monoclonal antibody regimen, comprising ALB9 aimed at CD24 and BL13 targeting CD21 (68, 69). Generally, the treatment regimen was well-received, with the primary side effects being temporary neutropenia of grade 3 or higher in 42% of cases, and grade 2 fever in 22% of patients during the initial infusion (68, 69). Instances of grade 3 sepsis, diarrhea, vomiting, and thrombocytopenia were each observed in one patient. The second trial was a phase 1/2 study conducted at a single institution, involving 36 individuals with primary hepatocellular carcinoma who had undergone surgical resection (70). The patients in this study were treated with adjuvant therapy, which included autologous transfusions of dendritic cells and cytokine-induced T cells, both loaded with the CD24 peptide (70). This treatment proved to be safe, with the most frequent side effect being a transient fever of less than grade 3, occurring in 19% of the participants. There were no reported adverse events of grade 3 or higher. After four years, the overall survival rates were 47% and 53% for patients who received the study treatment two and four times, respectively (70).

**Figure 4:**
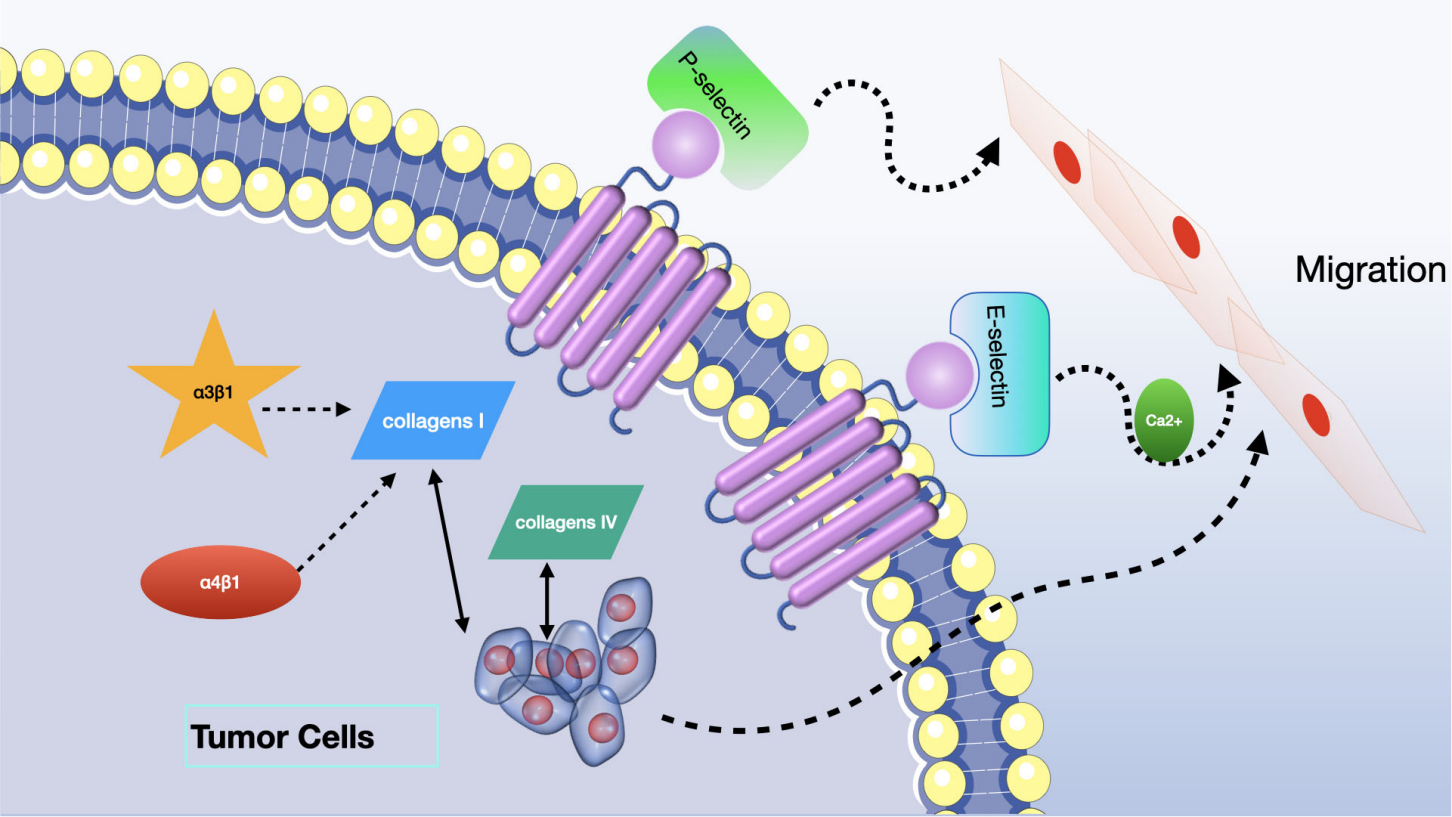
CD24 enhances tumor metastasis by either binding to selectin molecules or activating pre-existing integrin subunits.

Though significant progress has been achieved in understanding the functions of CD24 in cancer, research on its role in neural cancers has been relatively limited. For example, while CD24 polymorphisms have been studied in experimental autoimmune encephalomyelitis (EAE) and various cancer types, their examination within the scope of neural cancers has been lacking. Gleaning knowledge from findings in various types of cancer, along with CD24’s complex involvement with cellular communication networks as previously mentioned, ought to provide a more profound comprehension of CD24’s function in the biology of neural tumors. The ability of CD24 on the cell surface to encourage the spread of cancer is associated with its interaction with P-selectin, which is found on stimulated platelets and the cells lining blood vessels (57) (see **Figure 4**).

CD24 is recognized as a potential indicator for CSCs or cells initiating brain tumors because it is found on the cell surface and is linked to the advancement of metastasis. In solid tumor CSCs of breast cancer, a CD44+/CD24− phenotype has been identified. These CD44+/CD24−/low breast CSCs have demonstrated increased tumorigenic capabilities (48, 71). Additionally, breast cancer cases exhibiting the CD44+/CD24−/low phenotype display poor prognosis and limited response to chemotherapy (72). However, identifying cancer stem cells in this manner has been met with challenges, including conflicting outcomes and considerable heterogeneity within and among various cancer subtypes (73).

## CD24 and non-neoplastic disorders

Although CD24 is best known for its involvement in cancer, it also plays a role in various nonneoplastic diseases. It has been implicated in inflammatory and autoimmune diseases, infectious diseases, and neurological disorders. Here, we discuss some of the key roles of CD24 in these nonneoplastic diseases.

### Autoimmune diseases

A decade ago, the initial connection between CD24 and autoimmune diseases surfaced when it was discovered that mice lacking CD24 displayed significant resistance to experimental autoimmune encephalomyelitis (22). CD24 is involved in the regulation of the immune response and has been implicated in the pathogenesis of autoimmune diseases. Clinical data provide substantial support for the association between CD24 and autoimmune diseases. CD24 polymorphisms are linked to the progression of autoimmune disorders, including systemic lupus erythematosus (SLE), multiple sclerosis and rheumatoid arthritis (22, 24, 74–76). Within the CD24 gene, there exists a single nucleotide polymorphism (SNP) denoted as P170, which results in a nonconservative alteration in the C-terminus of the mature CD24 protein, either as Alanine (A, P170C) or Valine (A, P170T).

*Zhou et al.* initially reported that the CD24V/V genotype was associated with an increased risk and progression of multiple sclerosis. They noted that the expression of CD24 on peripheral blood T cells was higher in CD24V/V patients compared to those with the CD24A/A genotype. This association was subsequently validated in a Spanish cohort, although contradictory data were reported by another group from two cohorts (77, 78). In the case of SLE, *Sanchez et al.* conducted a study involving three Caucasian cohorts from Spain, Germany, and Sweden. They discovered that the prevalence of the CD24V/V genotype was elevated in SLE patients in the Spanish cohort, though this trend was not observed in the German or Swedish cohorts 24). For rheumatoid arthritis, the CD24V/V genotype was found to be more common among patients when a large screening of over a thousand rheumatoid arthritis patients and eight hundred healthy individuals was conducted (76). A similar association was observed in giant cell arthritis (79).

Additionally, there are three other polymorphisms located in the CD24 mRNA long UTR, namely P1056, P1527, and P1626. Among these, the dinucleotide deletion of P1527 has the capacity to destabilize CD24 mRNA and, significantly, this deletion offers protection against both multiple sclerosis and SLE (80).

Multiple sclerosis stands as the most prevalent autoimmune disorder within the central nervous system, characterized by persistent inflammation and extensive damage involving the loss of myelin and axons. The initial onset of MS involves the infiltration of the CNS by self-targeting immune cells, specifically T cells. The incidence of MS has been associated with both environmental and genetic factors (81, 82). CD24, which is under developmental regulation in T cells (7), serves as a co-stimulatory molecule that enhances T cell activation (83). In the experimental model for MS, experimental autoimmune encephalomyelitis (EAE), CD24 is essential for autoreactive T cells as well as resident CNS lymphocytes (22, 75).

The precise mechanism through which CD24 influences autoimmune diseases remains to be fully understood. In addition to its role as a costimulatory factor, CD24 serves as a genetic checkpoint in the context of T-cell homeostatic proliferation, especially in lymphogenic hosts. Lymphopenia, a common occurrence in autoimmune diseases, triggers T-cell homeostatic proliferation. It has been established that CD24 expressed on T cells plays a crucial role in this process (84, 85). Additionally, considering that CD24 influences the efficiency of clonal deletion, it is plausible that mice carrying a specific mutation in CD24 could exhibit a decreased presence of high-affinity autoreactive T cells (86).

### Inflammation diseases

Inflammation is a natural immune response to infections and tissue damage. It is triggered by various agents, which can be categorized into two main groups. The primary and most influential category includes molecular patterns related to pathogens, known as PAMPs, while the secondary category, which is of lesser importance, contains molecular patterns associated with damage, known as DAMPs (87, 88).

**Figure 5:**
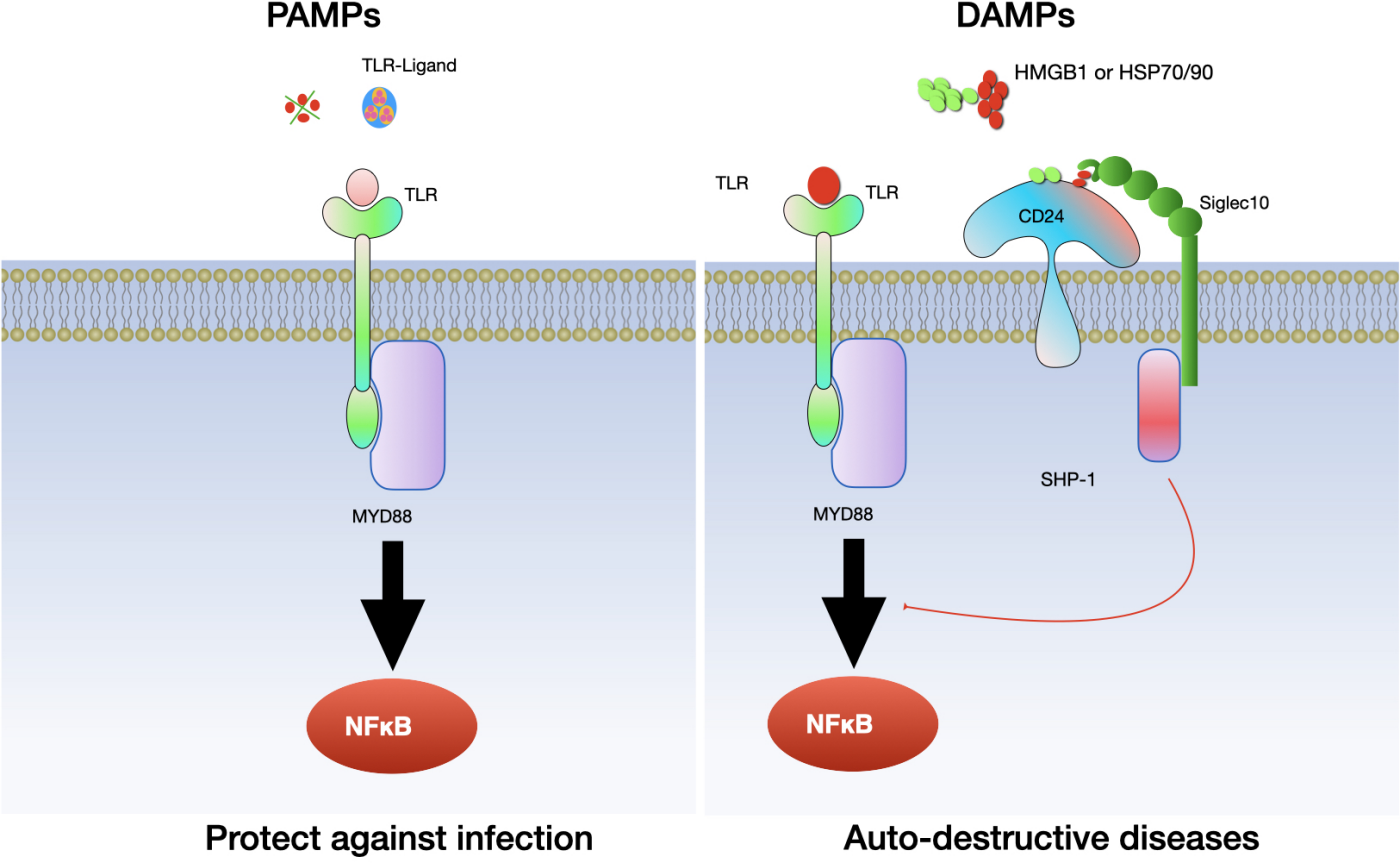
The interaction between CD24 and Siglec 10/G specifically inhibits the inflammatory reaction to tissue damage. CD24 binds with Siglec-10 to suppress inflammatory responses triggered by danger-associated molecular patterns (DAMPs), while it does not affect those elicited by pathogen-associated molecular patterns (PAMPs). Toll-like receptors (TLRs) are involved in this process.

In the context of inflammatory diseases, CD24 has a multifaceted role, acting as both a facilitator and regulator of inflammation. Its function is highly dependent on the context of the disease and the type of immune cells involved. In initial research, it was observed that the interaction between CD24 and Siglec G/10 plays a role in controlling the inflammatory response to DAMPs but not PAMPs. Subsequently, more and more evidence confirmed the relationship between CD24 and several DAMPs, including heat-shock proteins (HSP), high mobility group box protein 1 (HMGB-1) and nucleolins (89, 90). CD24 interacts with SiglecG in mice and Siglec10 in humans to specifically suppress the host’s reaction to tissue injury. Notably, this mechanism does not interfere with the host’s response to PAMPs (91). As a result, the CD24-SiglecG pathway is suggested to have the ability to differentiate between DAMPs and PAMPs (91) (see **Figure 5**).

Nevertheless, the response of the host to infectious pathogens can be affected by this interaction, since most infections lead to cell death and could provoke inflammatory reactions via DAMPs. Additionally, numerous pathogens have been discovered to interrupt the CD24-Siglec G/10 connection, either by diminishing the expression of Siglec G/10 or by removing sialic acids from CD24 (25, 92). CD24 has been found to be upregulated in response to viral and bacterial infections, and CD24-deficient mice have been shown to be more susceptible to infection (93–97). Preclinical studies have demonstrated that CD24Fc was effective in protecting non-human primates from acquired immunodeficiency syndrome (AIDS) caused by the simian immunodeficiency virus (98, 99). Human CD24-Fc has been effectively created and experimented with, particularly in rhesus monkeys afflicted with chronic immune issues and inflammation resulting from HIV-1/SIV infection. The treatment was well-received in these tests, suggesting it might help slow the progression to AIDS in SIV-infected primates.

CD24-Fc shows potential as an innovative approach to manage the immune response in diseases marked by persistent immune activation and systemic inflammation. Its effectiveness is currently being further explored in various clinical trials for immune-related conditions such as graft-vs-host disease and other similar disorders. It has been implicated in the regulation of immune responses to infections, including the production of cytokines and chemokines and the recruitment of immune cells to the site of infection (25, 100–103).

CD24 could be proposed to discriminate between DAMPs and PAMPs and deeper understanding CD24-Siglec10 interaction pathways may lead to the development of targeted therapies for managing inflammatory conditions.

### Covid-19

Based on the positive results in non-human primates, as well as the safety and clinical performance of CD24Fc in healthy volunteers and patients undergoing bone marrow transplantation, OncoImmune, Inc. initiated a phase 3 clinical trial at nine medical centers in the United States. This study is designed to evaluate the safety and clinical effectiveness of CD24Fc in hospitalized COVID-19 patients requiring oxygen support. The main goal is to measure the duration until clinical improvement, which is marked by the patient’s shift from needing oxygen support to breathing without it over a 28-day observation period 154,.

The data from this clinical trial indicates that CD24Fc is well-received and markedly hastens the rate of clinical recovery by over 60% in hospitalized patients with COVID-19 who require oxygen support. Biomarker studies have revealed that CD24Fc consistently suppresses the inflammatory response in COVID-19 patients. Overall, these results indicate that focusing on inflammation due to tissue damage might provide a treatment possibility for COVID-19 patients in the hospital (100).

In line with the clinical outcomes of CD24Fc, it was observed that HMGB1 (High Mobility Group Box 1) is elevated in the plasma of COVID-19 patients (104, 105). Furthermore, RNA sequencing analysis of lung tissue from both healthy individuals and severe COVID-19 patients showed a selective reduction in SIGLEC10 mRNA expression without affecting the expression of other SIGLECS (92). More recently, a non-randomized study by *Shapira et al.* suggested that Exo-CD24, which includes CD24-containing exosomes, appeared to reduce inflammatory markers and cytokines/chemokines while expediting the recovery of hospitalized COVID-19 patients (106).

### Neurological disorders

CD24 has been found to promote axonal growth and myelination, and CD24-deficient mice have been shown to have impaired neural development (32, 107, 108). Like numerous other surface antigens present on neural cells, CD24 holds significance in cellular communication and function, particularly in processes such as neural migration, the extension of neurites, and neurogenesis (109). Studies indicate dynamic expression of CD24 during the neural development of rodents, and at least one study has suggested transient expression of CD24 during human development (110–112). These findings also emphasize that CD24 could undergo transcriptional activation in postmitotic neurons during the migration phase but is typically lost once a more mature cytoarchitectural context is established. *Calaora* and colleagues showed that the expression of mCD24 is maintained in certain areas of adult mice that are involved in the secondary formation of neurons (110). Notably, CD24 expression endures into adulthood within the rostral migratory stream, the pathway used by newly formed neurons toward the olfactory bulb, as well as in the dentate gyrus of the hippocampus. These results support the notion that CD24 is present during stages of neuronal migration and the formation of neuronal connections. The observations are consistent with the idea that CD24 functions as a glycoprotein that plays a role in directing neuronal migration and the formation of synapses. Furthermore, CD24 expression was observed in non-neuronal ependymal cells that possess cilia and line the ventricles (113). The precise degree to which CD24’s glycans may distinctively guide the movement of neurons and the creation of connections during this stage of development is still a matter to be determined through research.

CD24 has also been implicated in the pathogenesis of neurological disorders, including neuronal injury, etc. CD24 can enhance neuronal regeneration in experimental subarachnoid hemorrhages (114). Astrocytes have the potential to alleviate neuronal damage by utilizing CD24 to inhibit NF-κB binding activity, thereby reducing the secretion of inflammatory factors subsequent to the HMGB1 challenge (see **Figure 5**). CD24 appears to enable T cells to evade clonal deletion, a process that deactivates self-reactive cells before maturation, but it does not influence the entry of self-reactive T cells into the CNS (86, 115). The expression of CD24 on resident CNS lymphocytes exacerbates the severity of EAE by boosting the activation of autoreactive T cells (75), crucial for their local multiplication (115). This interaction does not seem to involve a like-to-like trans interaction between CD24 molecules (116). Overall, the cumulative evidence suggests that CD24 expression encourages the development of EAE, and potentially MS, following initiation by various other contributing factors (117).

### Metabolic disorder

Metabolic disorders such as obesity, dyslipidemia, diabetes, nonalcoholic fatty liver disease, and nonalcoholic steatohepatitis have significantly emerged as a major global health concern (118). Metaflammation, characterized as a persistent low-grade inflammatory state in metabolic tissues, stands as a significant hallmark of metabolic disease (119). This chronic state of tissue inflammation involves the infiltration and activation of immune cells along with elevated levels of inflammatory cytokines, resulting in impaired insulin signaling and disruption of systemic metabolic balance (120). Several inflammatory signaling pathways like JNK and IKK, alongside inflammatory cytokines such as TNF-α and IL-1β, have been implicated in the development of metabolic diseases (121–124) Despite compelling evidence linking chronic inflammation to obesity, the mechanisms underlying the initiation and control of metaflammation during obesity remain inadequately understood.

**Figure 6:**
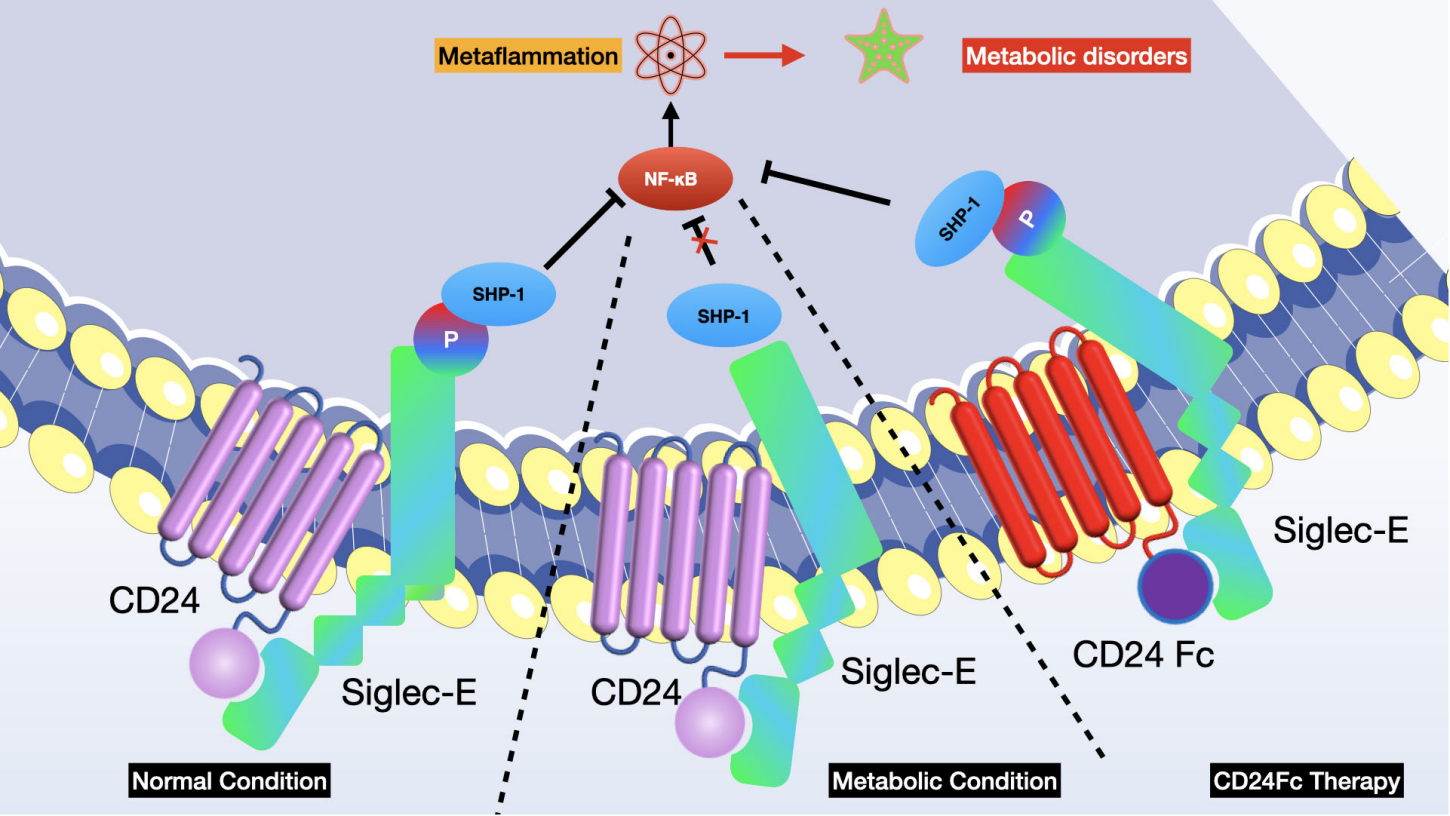
Disruption of the CD24-Siglec-E axis worsens, whereas CD24Fc treatment improves, metabolic disorders associated with obesity. Sialylated CD24 triggers the recruitment of SHP-1 to Siglec-E, thereby reducing metaflammation. In the initial phase I clinical trial conducted on humans, a sole administration of CD24Fc proved safe and well-received among healthy individuals.

*Yang L et al.* identified that the disruption of the CD24-Siglec-E interaction exacerbates metabolic disorders associated with obesity, while therapy involving CD24Fc shows improvement (26). The recognition of CD24 by Siglec-E through sialoside-based interactions negatively regulates metaflammation and offers protection against metabolic syndrome. Clinical studies on CD24Fc confirm the significance of this pathway in human lipid processing and inflammatory responses. These discoveries highlight the pivotal inhibitory function of the CD24-Siglec-E axis in metabolic dysfunctions and metaflammation, offering a potential immunotherapeutic approach for conditions like obesity, dyslipidemia, insulin resistance, and nonalcoholic steatohepatitis. They also evaluated the role of CD24-Siglec pathway in the development of metabolic syndrome and demonstrated that CD24 deficiency aggravates metabolic disorder in mice. To pinpoint the CD24 receptor involved in metabolic regulation, researchers employed a genetic strategy to ascertain if mutations in any *Siglec* gene could replicate the metabolic characteristics observed when CD24 is removed. They finally found that Siglec-E signaling is required for CD24-mediated protection against metabolic disorder and CD24-Siglec-E axis represses metaflammation to ameliorate metabolic disorder. Given that CD24Fc is undergoing clinical development for several human diseases, the recent study offers a promising therapeutic strategy for treating metabolic diseases by strengthening sialoside-based pattern recognition (see **Figure 6**).

## Conclusion

CD24 has emerged as a promising therapeutic target in cancer due to its significant role in the disease. Researchers have extensively investigated the use of antibodies that target CD24 for cancer treatment. While preclinical studies exploring CD24 antibody-based therapies have been conducted and reviewed previously (**Table 1**) (125), it’s worth noting that as of now, there have been no clinical trials targeting CD24 in cancer treatment. While the role of CD24 in cancer has garnered significant attention, its importance extends to various other physiological and pathological processes, including autoimmune diseases, infectious disorders, Covid-19, neurological disorders, metabolic disorders and more. Further research into CD24 functions in these areas may reveal new insights and potential therapeutic applications beyond cancer.

**Table 1:** Clinical trials with agents targeting CD24.

Trial Identifier	Inclusion	Agent	Phase	Status	Primary Outcome	Enrollment	Institute
NCT03960541	HIV Infections Dyslipidemias	Efprezimod alfa	II	Terminated	Decrease LDL and Inflammation	8	University of Maryland Baltimore
NCT04747574	SARS-CoV-2	EXO-CD24	I	unknown	N/A	35	Tel Aviv Medical Center
NCT04060407	Metastatic Melanoma	CD24Fc	Ib/II	withdrawn	N/A	0	Huntsman Cancer Institute
NCT04552704	Advanced Malignant Solid Neoplasm	CD24Fc	I/II	Terminated	N/A	3	University of California Davis
NCT02663622	GVHD Leukemia	Efprezimod alfa	II	Completed	Strong protection against GVHD	44	Indiana University School of Medicine etc
NCT04907422	Carcinoma Ex Pleomorphic Adenoma of Salivary Glands Pleomorphic Adenoma of Salivary Glands	CD24-Gold Nanocomposite	II	Completed	N/A	60	October 6 University
NCT04317040	Coronavirus Disease 2019 (COVID-19)	Efprezimod alfa	III	completed	clinical improvement was accelerated in the CD24Fc group	234	Baptist Health Research Institute etc.
NCT04902183	Coronavirus Disease 2019 (COVID-19)	exosomes overexpressing CD24	II	recruiting	N/A	90	General Hospital of Athens Attikon University Hospital
NCT01214512	Colorectal Cancer	Micromedic CD24	I	completed	N/A	229	Bat Yamon Gastroentrology Clinic etc.
NCT04095858	GVHD AML	CD24Fc	III	Terminated	N/A	11	City of Hope etc.

To enhance patient outcomes, it is imperative to tackle the issue of drug resistance mediated by CD24 and prolong patient survival. Nevertheless, the exact mechanisms through which CD24 contributes to drug resistance in tumor cells remain incompletely elucidated, involving a complex interplay of diverse pathways. Clinically, it requires additional assessment to determine how to integrate phagocytosis checkpoint inhibitors and/or activators into the existing framework of immunotherapy. In addition, investigating the potential for combining CD24-targeted immunotherapy with other treatments, such as checkpoint inhibitors or chemotherapy, is a critical area of research. Determining the synergistic effects and potential toxicities of combination therapies is essential. Furthermore, designing well-controlled clinical trials with appropriate endpoints and patient cohorts is essential for robustly evaluating the efficacy of CD24-targeted immunotherapy. Also, Assessing the safety profile of CD24-targeted immunotherapy is crucial. Understanding potential side effects and their management is essential to ensure that the treatment does not cause significant harm to patients. Moreover, identifying predictive biomarkers that can help select patients who are most likely to respond to CD24-targeted immunotherapy is an important consideration. This will help in patient stratification and personalized treatment approaches.

In summary, while CD24-targeted immunotherapy holds promise in the treatment of cancer and non-neoplastic diseases, numerous questions related to its clinical application, efficacy, safety, and combination strategies still need to be addressed through rigorous research and clinical trials.

## Author contributions

HW: Software, Writing – original draft. PS: Writing – review & editing, Supervision, Investigation. XS: Data curation, Writing – original draft. YL: Resources, Writing – original draft. HX: Supervision, Writing – review & editing. HZ: Conceptualization, Funding acquisition, Investigation, Supervision, Writing – original draft, Writing – review & editing.
